# The pan-cancer landscape of aldo-keto reductase1B10 reveals that its expression is diminished in gastric cancer

**DOI:** 10.3389/fimmu.2024.1488042

**Published:** 2024-12-06

**Authors:** Anqi Wu, Hao Li, Mengnan Gao, Juan Liang, Jiaqi Huang, Jaume Farrés, Deliang Cao, Guoqing Li

**Affiliations:** ^1^ Department of Clinical Research Center, The Second Affiliated Hospital, Hengyang Medical School, University of South China, Hengyang, China; ^2^ Hunan Province Key Laboratory of Basic and Clinical Pharmacological Research on Gastrointestinal Tumors, The Second Affiliated Hospital, Hengyang Medical School, University of South China, Hengyang, China; ^3^ Department of Pathology, The Second Affiliated Hospital, Hengyang Medical School, University of South China, Hengyang, China; ^4^ Department of Gastroenterology, The Second Affiliated Hospital, Hengyang Medical School, University of South China, Hengyang, China; ^5^ Department of Biochemistry and Molecular Biology, Universitat Autònoma de Barcelona, Barcelona, Spain; ^6^ Hunan Province Key Laboratory of Cancer Cellular and Molecular Pathology, Hengyang Medical School, Cancer Research Institute, University of South China, Hengyang, China

**Keywords:** AKR1B10, pan-cancer, progression, tumor microenvironment, immunity

## Abstract

**Introduction:**

Aldo-keto reductase 1B10 (AKR1B10) is a multifunctional enzyme, which is important in cancer development and progression, but the landscape of AKR1B10 in pan-cancers and in tumor microenvironment is unclear.

**Method:**

This study integrated the sequencing data of 33 cancer types, including gastric cancer, from TCGA project to explored the expression pattern and genetic and epigenetic alterations of AKR1B10. The association of AKR1B10 expression with clinical progression of cancers was evaluated by Kaplan-Meier analysis; the potential role of AKR1B10 in tumor microenvironment (TME) and immune-related gene expression were analyzed by PURITY, ESTIMATE, TIMER and CIBERSORT algorithms. The expression of AKR1B10 and immune cell markers in gastric cancer were evaluated with multiplex immunofluorescence staining.

**Result:**

Results indicated that AKR1B10 was highly expressed in the gastrointestinal tract in health donors, but the expression of AKR1B10 was significantly changed in most of cancer types, which may be ascribed to DNA methylation in its promoter. The AKR1B10 expression in cancers and its value in disease progression was bidirectional and functionally enriched in metabolism in pan-cancers. In tumor microenvironment, AKR1B10 was significantly correlated with immune cell infiltrations and immune gene expression. In the stomach, along with the diminishing of AKR1B10 expression, CD68+ macrophage increased and CD19+ B cell decreased in gastric cancer.

**Discussion:**

These data indicates that AKR1B10 may be an important factor in the development and progression and a potential therapeutic target for multiple cancers, but plays as a protector in the gastric tissues.

## 1 Introduction

Aldo-keto reductase 1B10 (AKR1B10), also known as aldose reductase-like 1 (ARL-1), is a member of aldo/keto reductase (AKR) superfamily. AKR1B10 along with AKR1B1 and AKR1B15 together belong to the human AKR1B subfamily. These genes cluster at chromosome 7q33, and share more than 60% amino acid sequence identity (1). AKR1Bs catalyze the reduction of aromatic and aliphatic aldehydes to alcoholic forms with NADPH as a co-enzyme (1–5). These enzymes show certain substrates overlaps, but with clear variation of the catalytic efficiencies. For instance, AKR1B10 specifically catalyzes retinal (all-trans-retinaldehyde) with high efficiency to retinol (3), but its glucose reductase activity (1–3), prostaglandin F synthase activity (6), 17β-hydroxysteroid dehydrogenase activity for estrone and 4-androstene-3,17-dione (4, 5) are relatively limited. AKR1B10 also efficiently reduces endogenous carbonyl compounds, including lipid peroxidation-derived cytotoxic aldehydes (2), isoprenyl aldehydes (7), and xenobiotics, such as drugs (8) and polycyclic aromatic hydrocarbon derivatives (9). The active site of AKR1B10 is the α/β-barrel tertiary structure which is consisted of 8 α-helices and β-sheets (10).

Under physiological conditions, AKR1B10 is constitutively abundant in small intestine and colon (1, 11), which can protect mucosa epithelial cells from lesions of the reactive aldehydes in digested food through reductive detoxification. Pathologically, abnormal AKR1B10 expression is associated with various skin diseases (12–14), precancerous diseases (15–22) and cancerous diseases. However, the role of AKR1B10 in cancer development and progression is complex. AKR1B10 expression is elevated and correlated with poor prognosis in several types of cancers like hepatocellular carcinoma (23), breast carcinoma (24–26), lung cancer (27–29) and oral cancer (30–32), but is quite opposite in colorectal cancer (33–37). AKR1B10 may promote cancer progression through fatty acid synthesis, but prevent cancer via regulating autophagy and FGF1. Therefore, a special function of AKR1B10 in an indicated type of cancer may be context-dependent. A comprehensive analysis of AKR1B10 in pan-cancers is certainly warranted.

In this study, we compared the expression of AKR1B10 in multiple types of normal tissues and cancers, evaluated the relationship of AKR1B10 expression with DNA mutation, DNA methylation, cancer clinicopathology, cancer microenvironment and immune cell infiltration, and survivals in pan-cancers. Our study for the first time presents a landscape of AKR1B10 in pan-cancers and discusses the context-dependent function of AKR1B10 in cancers.

## 2 Material and methods

### 2.1 Data collection

All of the data analyzed in this article were public available. The RNA sequencing data of health tissues was downloaded from Genotype-Tissue Expression (GTEx) portal (https://www.gtexportal.org/) which collects organs from autopsies. The RNA sequencing data, simple nucleotide variations, copy number variations, as well as clinical data of TCGA projects were downloaded from GDC portal (https://portal.gdc.cancer.gov/), which contains datasets of adrenocortical carcinoma (ACC), bladder urothelial carcinoma (BLCA), breast invasive carcinoma (BRCA), cervical squamous cell carcinoma and endocervical adenocarcinoma (CESC), cholangiocarcinoma (CHOL), colon adenocarcinoma (COAD), lymphoid neoplasm diffuse large B-cell lymphoma (DLBC), esophageal carcinoma (ESCA), glioblastoma multiforme (GBM), head and neck squamous cell carcinoma (HNSC), kidney chromophobe (KICH), kidney renal papillary cell carcinoma (KIRP), kidney renal clear cell carcinoma (KIRC), acute myeloid leukemia (LAML), brain lower grade glioma (LGG), liver hepatocellular carcinoma (LIHC), lung adenocarcinoma (LUAD), lung squamous cell carcinoma (LUSC), mesothelioma (MESO), ovarian serous cystadenocarcinoma (OV), pancreatic adenocarcinoma (PAAD), pheochromocytoma and paraganglioma (PCPG), prostate adenocarcinoma (PRAD), rectum adenocarcinoma (READ), sarcoma (SARC), stomach adenocarcinoma (STAD), skin cutaneous melanoma (SKCM), testicular germ cell tumors (TGCT), thyroid carcinoma (THCA), thymoma (THYM), uterine corpus endometrial carcinoma (UCEC), uterine carcinosarcoma (UCS), uveal melanoma (UVM).

The immunohistochemistry (IHC) results of AKR1B10 in cancers and normal tissues were downloaded from the Human Protein Atlas (HPA) database (https://www.proteinatlas.org/). STAD enrolled samples from stomach (T-6300), stomach upper (T-62350) and stomach lower (T-63700) Adenocarcinoma; COAD enrolled samples from colon (T-67000) adenocarcinoma; LIHC enrolled samples from liver (T-56000) hepatocellular carcinoma; PAAD enrolled pancreas (T-59000) adenocarcinoma; UCEC enrolled samples from endometrium (T-84000) adenocarcinoma; CESC enrolled samples from cervix (T-83000) squamous cell carcinoma.

The validation datasets (GSE107850, GSE7696, GSE41272, GSE8894 and GSE57303) were available in gene expression omnibus (GEO) database (https://www.ncbi.nlm.nih.gov/geo/).

### 2.2 Analysis of AKR1B10 expression and clinical features

The association of AKR1B10 expression with stages or grades in each cancer from TCGA database was analyzed by TISIDB (http://cis.hku.hk/TISIDB/), which is an integrated repository portal for tumor-immune system interactions.

The prognosis information including overall survival (OS), progression-free interval (PFI) and disease-free interval (DFI) from clinical follow-up data were integrated with AKR1B10 expression from RNA sequencing data. The cutoff value of AKR1B10 expression was determined by the optimal calculation result. The prognostic value of AKR1B10 was determined by Kaplan-Meier analysis.

### 2.3 Mutation analysis

The frequency and site of mutations (missense mutations, nonsense mutations, frame shift inserts, frame shift deletions and splice sites) in each cancer based on the simple nucleotide variation dataset processed by MuTect2 software (38) were integrated with AKR1B10 protein domain information acquired from maftools (version 2.2.10) in R software. The tumor mutation burden (TMB)scores were calculated by tmb function of maftool.

The copy number variation (CNV) datasets processed by GISTIC software (39) were integrated with log2(x+0.001) transformed AKR1B10 expression from RNA-seq datasets. A total of 26 types of cancers were analyzed with R software (version 3.6.4) when cancers with sample number less than 3 were excluded.

### 2.4 Visualization of methylation data and single cell RNA sequencing data

The correlation between methylation of AKR1B10 promoter and FPKM in each cancer was analyzed by DNMIVD (40) which is an interactive visualization database for DNA methylation profiles of diverse human cancers including TCGA databases.

The expression of AKR1B10 among different organs and cell types from single cell RNA sequencing were interrogated and visualized by Human Cell Landscape (http://bjs.zju.edu.cn/HCL/) (41), which contains 60 human tissue samples from 8 systems.

### 2.5 Microenvironment analysis

The stromal, immune and ESTIMATE scores of each cancer sample were calculated by ESTIMATE package (version 1.0.13) in R software based on the gene expression matrix after data normalization and transformation mentioned above. While the purity and homologous recombination deficiency (HRD) data of each cancer were downloaded from previous published literature (42). The expression of AKR1B10 was integrated with purity, stromal scores, immune scores, ESTIMATE scores, and HRD data, and then the correlation was analyzed with corr.test function of psych package (version 2.1.6) in R software.

### 2.6 Immune cell infiltration analysis

Normalized pan-cancer data were annotated with gene symbols, and the expression was transformed with log2(x+0.001). The infiltration scores of B cells, T cells CD4, T cells CD8, neutrophils, macrophages and DCs in cancers were calculated with TIMER (43) algorithm from IOBR package (version 0.99.9) (44) in R software. Moreover, the infiltration scores of B_cell_naive, B_cell_memory, plasma_cells, T_cells_CD8, T_cells_CD4_naive, T_cells_CD4_memory_resting, T_cells_CD4_memory_activated, T_cells_follicular_helper, T_cells_regulatory_(Tregs), T_cells_gamma_delta, NK_cells_activated, Monocytes, Macrophages_M0, Macrophages_M1, Macrophages_M2, Dendritic_cells_resting, Dendritic_cells_activated, Mast_cells_resting, Mast_cells_activated, Eosinophils and Neutrophils in cancers were determined by deconvo_CIBERSORT (45) algorithm from IOBR package in R software. The association between AKR1B10 expression and immune cell infiltration scores in each cancer was analyzed with corr.test function of psych package (version 2.1.6).

### 2.7 Correlation of AKR1B10 with immune related gene expression

The expression of 41 chemokine genes, 18 chemokine receptors, 21 MHC genes, 24 immunoinhibitors, 46 immunostimulators and 24 immune checkpoint inhibitors (42) was extracted from normalized pan-cancer RNA-seq data, the correlation of which with AKR1B10 in each cancer was analyzed with corr.test function of psych package (version 2.1.6) after log2(x+0.001) transformed.

### 2.8 Functional analysis

The function of AKR1B10 in pan-cancers was analyzed with gene set enrichment analysis (GSEA) software (version 4.2.3). RNA-seq data of 33 cancers from TCGA project were loaded into GSEA software. C2.cp.kegg.v7.5.1.symbols was chosen as a gene set database, and the number of permutations was set to 1000. The no_collapse was used to gene symbols, and phenotype was set for permutation type. Enriched result lists were first filtered with threshold of |NES|≥1.5, NOM p-value<0.05, and FDR q-value<0.25. Filtered results were further presented with a heatmap of NES.

### 2.9 Multiplex immunofluorescence staining

Gastric cancer tissue microarray (A962) was purchased from WeiAoBio (Shanghai, China). Multiplex immunofluorescence staining procedure on paraffin-embedded tissue section was followed by the instruction of supplier (Absin, Cat No. abs50013). In briefly, the tissue section was deparaffinize, rehydration, antigen retrieval by microwave, serum blocking, primary antibody incubation, secondary antibody incubation, fluorescence staining signal amplification (multiple rounds), staining nuclear with DAPI and mounting. The primary antibodies were AKR1B10 (Abnova, Cat No. H00057016-M01, 1:200), CD68 (Absin, Cat No. 171440, 1:200), CD19 (Abcam, Cat No. 134114, 1:100), PanCK (Absin, Cat No. 123684, 1:400). The staining with the horseradish peroxidase conjugated secondary antibody and the TSA tyramide signal amplification (520-TSA, 620-TSA, 570-TAS, 690-TSA) were supplied within the staining kit. Multispectral images were analyzed. The percentage of positive staining cell was calculated as number of positive cells divide total cell number (P). For the intensity (I), extensive staining was recorded as 3, moderate staining was recorded as 2, weak staining was recorded as 1. IHC score=∑(P×I)=(weak percentage×1)+(moderate percentage×2)+(strong percetage×3) .

### 2.10 Statistical analysis

The difference of AKR1B10 expression between cancers and corresponding normal tissues was analyzed by unpaired t-test. The significance between two groups was analyzed by unpaired Wilcoxon Rank Sum and Signed Rank tests. The significant difference among multiple groups was analyzed by the Kruskal test. All of the correlations were presented as spearman’s correlation coefficient.

## 3 Result

### 3.1 Tissue-specific expression of AKR1B10

**Figure 1:**
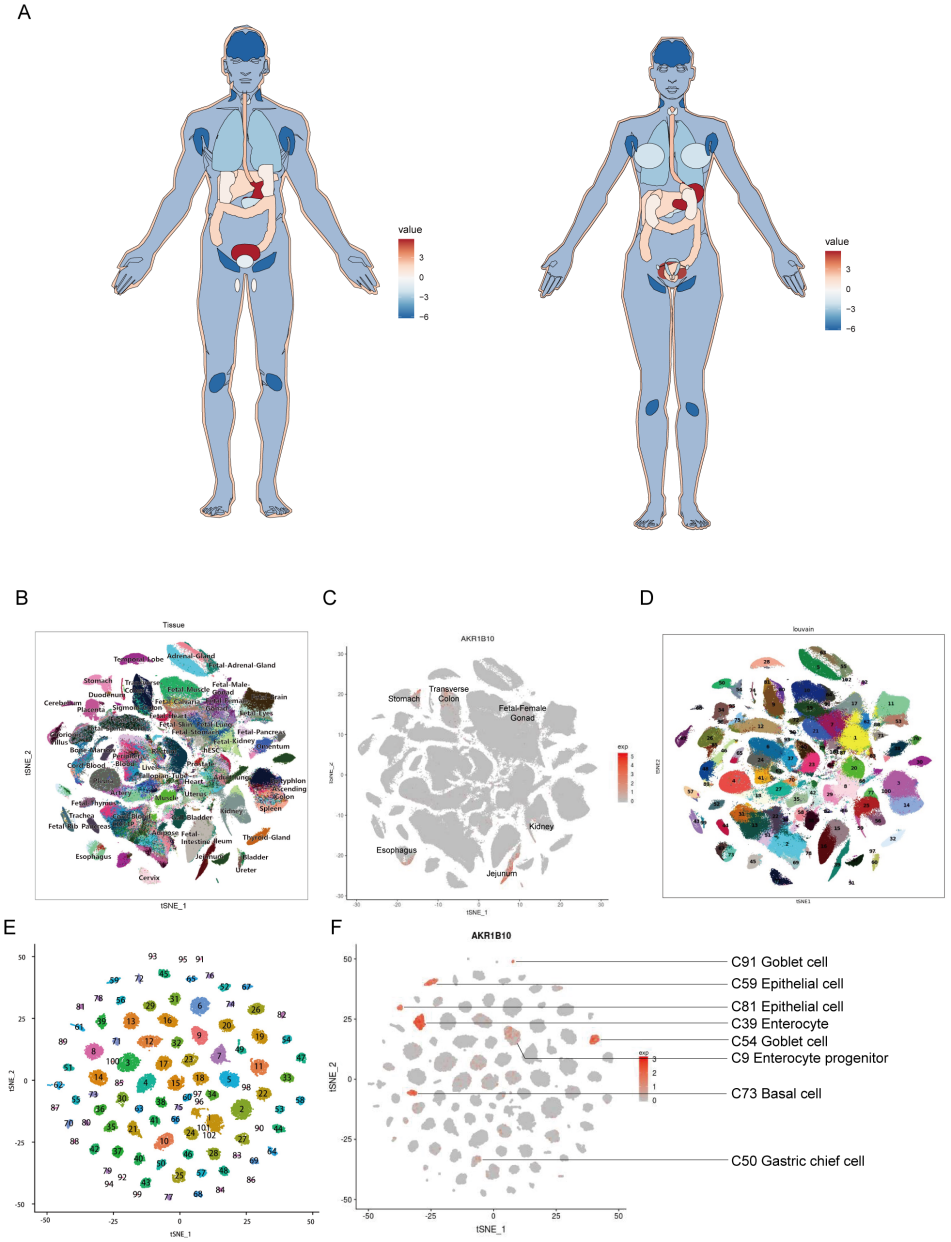
Expression landscape of AKR1B10 in normal tissues. *AKR1B10* expression in various organs based on RNA sequencing from GTEx databases, the color represents expression of AKR1B10 **(A)**. *AKR1B10* expression in various organs and cell types basing on single-cell RNA sequencing from HCL database **(B–F)**. The cell clusters basing on organs, and label represents name of each organ **(B)**. The cell cluster showed AKR1B10 expression in different organs **(C)**. The cell cluster showed relationship between organs and cell types **(D)**. The cell cluster basing on cell types, and label of cell type were indicated in separate **Supplementary Table 1** **(E)**. The cell cluster showed AKR1B10 expression in different cell types **(F)**. The color key represents expression of AKR1B10 **(D, F)**.

To demonstrate the expression of AKR1B10 in human normal tissues, we draw an anatomy map based on AKR1B10 expression from GTEx databases. The result showed that the AKR1B10 highly expressed in stomach and bladder, followed by esophagus, colon, liver, skin and adrenal gland, and AKR1B10 is barely expressed in brain and lymph node (**Figure 1A**). Overall, the gastrointestinal system showed a high-medium AKR1B10 expression, while development gastrointestinal system, genitourinary system, and gynecological system showed a medium-low AKR1B10 expression. The single-cell RNA sequencing (ScRNA Seq) data from Human Cell Landscape (HCL) also showed that the AKR1B10 is highly expressed in the jejunum, stomach, esophagus and transverse-colon (**Figures 1B, C**).

### 3.2 Aberrant AKR1B10 expression in pan-cancers

**Figure 2:**
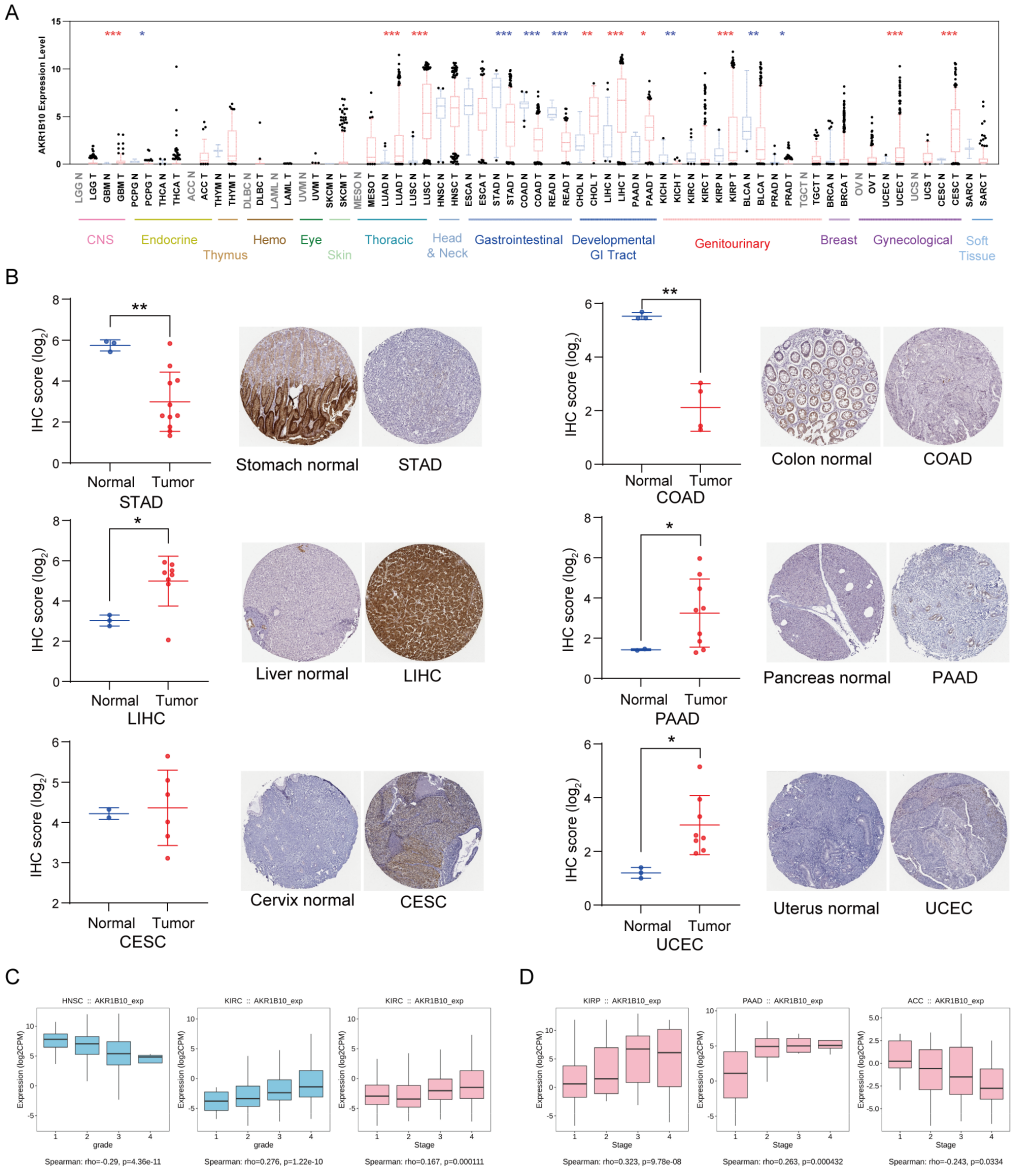
Aberrant expression of AKR1B10 in cancers. *AKR1B10* mRNA levels in 33 cancers and their corresponding para-cancer tissues from TCGA databases **(A)**. AKR1B10 protein expression in cancers from HPA databases by semi-quantification **(B)**. The association of *AKR1B10* expression with grades in HNSC and KIRC **(C)**. The association of *AKR1B10* expression with stages in KIRC, KIRP, PAAD and ACC **(D)**. * p < 0.05, ** p < 0.01, *** p < 0.001.

AKR1B10 expression is significantly altered in various types of cancers. In general, AKR1B10 mRNA down-regulated in most of gastrointestinal cancer, genitourinary cancer and PCPG, but up-regulated in most of developmental gastrointestinal cancer, some of gynecological cancer, glioma and lung cancer when compared with normal tissues from TCGA databases (**Figure 2A**). Consistently, AKR1B10 protein decreased in STAD and COAD, but increased in LIHC, PAAD, UCEC and CESC when compared with their individual normal tissues (**Figure 2B**). Moreover, we analyzed the association of AKR1B10 mRNA expression with clinical features. The results showed AKR1B10 mRNA expression was significantly correlated with grades of HNSC and KIRC (**Figure 2C**). AKR1B10 was also significantly correlated with stages of KIRC, KIRP, PAAD and ACC (**Figure 2D**).

**Figure 3:**
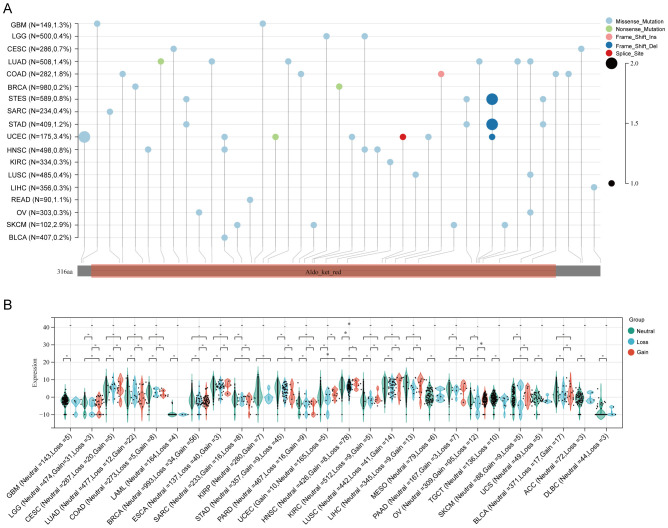
AKR1B10 gene mutations in various types of cancers. *AKR1B10* gene mutation landscape in cancers; the color represents different mutation types, and the size key represents frequency of mutation occured in each specific site **(A)**. The relationship between *AKR1B10* expression and CNV in cancers **(B)**. * p < 0.05.

**Figure 4:**
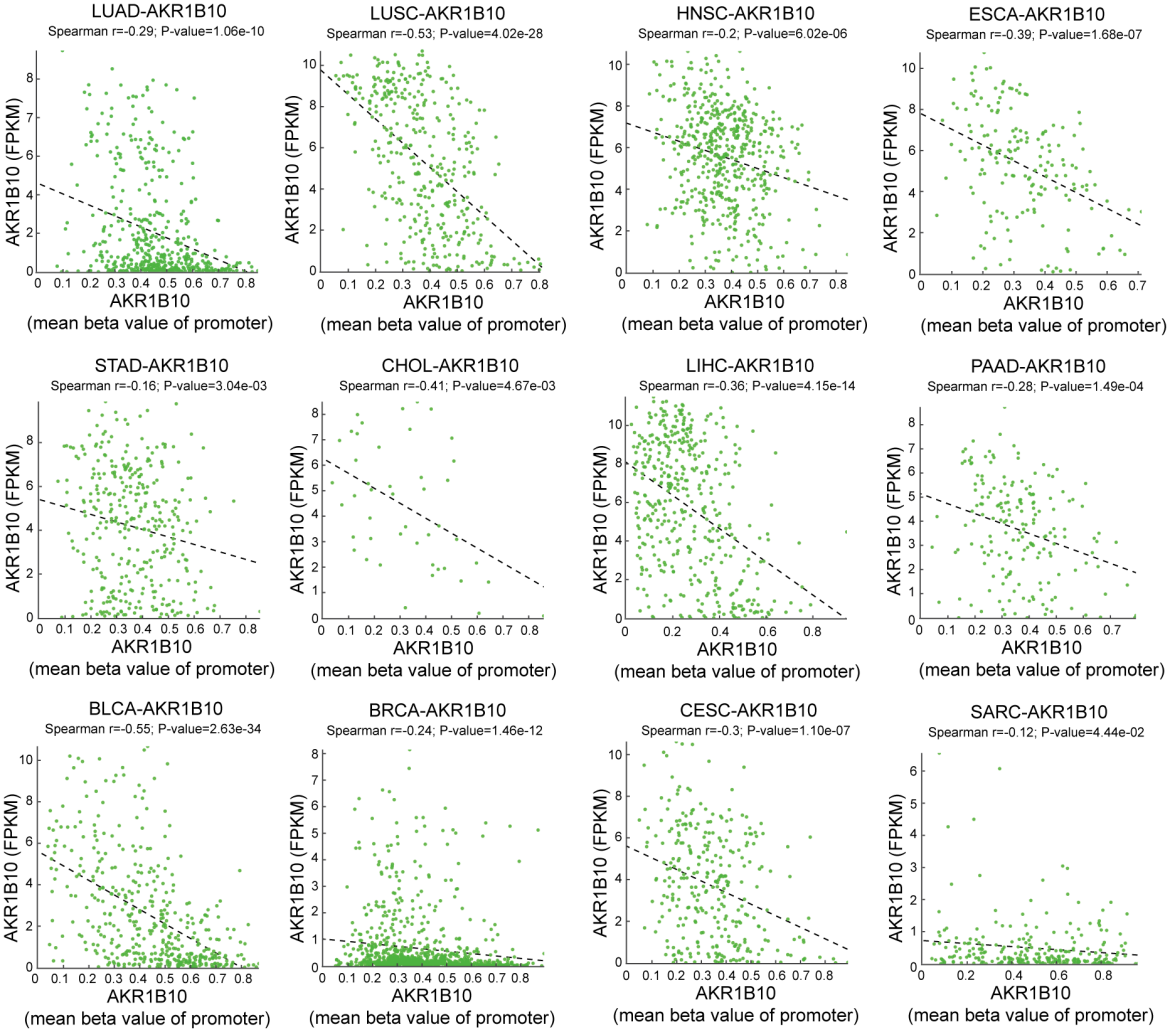
The correlation of AKR1B10 mRNA level and methylation levels. Correlation of AKR1B10 expression (FKPM) and AKR1B10 promoter methylation (mean beta value of promoter) in various cancers. The spearman correlation efficiency (r) and *p* value are indicated in each plot.

Both genetic and epigenetic alteration can result in gene expression change. Thus, we analyzed the relationship of DNA mutation, copy number variation (CNV) and DNA methylation with AKR1B10 expression. Mutation analysis indicated that scatter missense mutations (41), nonsense mutations (3), frame shift inserts (1), frame shift deletions (3) and splice sites (1) were observed within the coding sequence of AKR1B10 in 17/33 cancers, of which UCEC had the highest incidence rate of 3.4% (**Figure 3A**). CNV analysis showed that there are no distinct change in AKR1B10 expression among different types of CNV in most cancers (**Figure 3B**). While the methylation analysis showed that AKR1B10 expression was significantly negatively associated with DNA methylation in 12 types of cancer. Specifically, LUSC, CHOL and BLCA showed a moderate correlation (0.4< r <0.6), while LUAD, HNSC, ESCA, LIHC, PAAD, BRCA and CESC showed a weak correlation (0.2< r <0.4) (**Figure 4**). Taken together, epigenetic might contribute more to the alteration of AKR1B10 expression in pan-cancer.

### 3.3 Potential role of AKR1B10 in tumor microenvironment

Tumor microenvironment (TME) is the non-cancerous cells surround a tumor including fibroblasts and immune cells, which are reported to play a role in tumor growth and drug resistance Purity and ESTIMATE (including stromal score, immune score and ESTIMATE score) algorithm are novel computational methods to infer tumor purity from bulk cell genomic analysis. Purity patterns differ by cancer type (**Supplementary Figure 1A**). High purity is correlated with shorter OS of PCPG, ACC, ESCA, LUAD, READ,CHOL, CESC and SARC. It is also correlated with longer OS of UVM, MESO, STAD, PAAD, KIRC, BLCA and TGCT (**Supplementary Figure 1B**). High stromal score is correlated with shorter OS in LGG,GBM, LAML, UVM, MESO, LUSC, STAD, COAD, KIRC, KIRP and BLCA. It is also correlated with longer OS in THCA, ACC, SKCM, LUAD, CHOL, LIHC and SARC (**Supplementary Figure 2**). High immune score is correlated with shorter OS in LGG, LAML, UVM, ESCA and KIRC. It is also correlated with longer OS in PCPG, THCA, ACC, SKCM, MESO, LUAD, HNSC, COAD, CHOL, LIHC, KICH, BRCA and CESC (**Supplementary Figure 3**). High ESTIMATE score is correlated with shorter OS in LGG, GBM, LAML, UVM, LUSC, ESCA, STAD, PAAD, KIRC, KIRP and TGCT. It is also correlated with longer OS in PCPG, THCA, ACC, SKCM, LUAD, CHOL, LIHC, CESC and SARC (**Supplementary Figure 4**).

**Figure 5:**
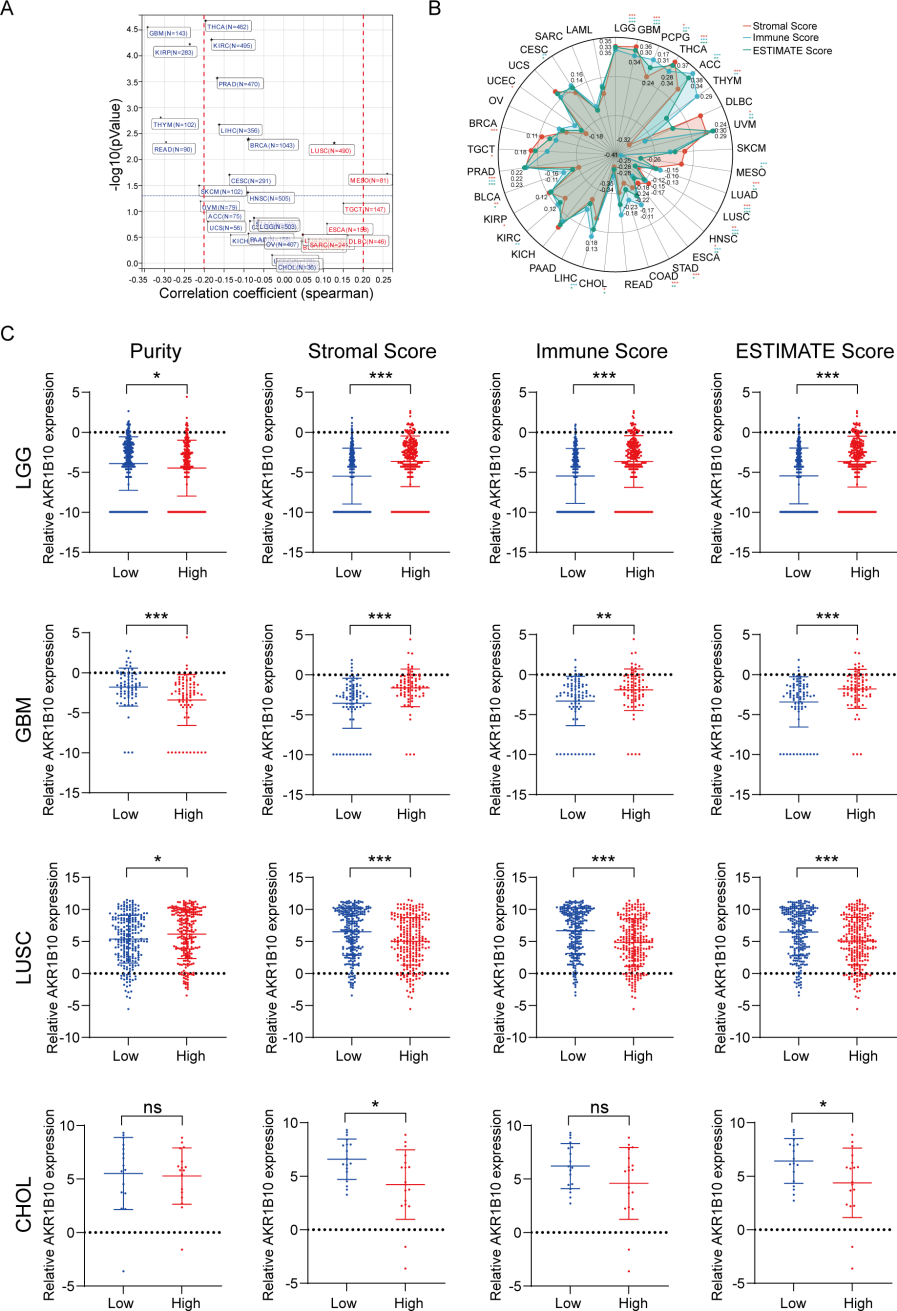
The association of AKR1B10 expression with TME. Scatter plot of *AKR1B10* correlation with tumor purity in pan cancer, tumors in red font indicates a positive correlation, while blue font indicates a negative correlation **(A)**. Radar plot of AKR1B10 correlation with microenvironment scores by the ESTIMATE algorithm **(B)**. The AKR1B10 expression in different group of LGG, GBM, LUSC and CHOL **(C)**. * p < 0.05, ** p < 0.01, *** p < 0.001; ns stands for not significant.

From the HCL, we knew the AKR1B10 expressed in the epithelial cells, goblet cells, basal cells, enterocytes and enterocyte progenitors, gastric chief cells and hepatocytes, as well as some T cells, B cells, fibroblasts and endothelial cells in normal tissues (**Figures 1D–F**). The function of AKR1B10 in cancer have attracted the attention of scientists, the role of AKR1B10 in TME remains elusive. Herein, we analyzed the association of AKR1B10 expression with tumor purity (**Figure 5A**). There were 7 cancers stood out, of which the MESO showed positive correlation (r>0.2), but the GBM, THYM, THCA, READ, SKCM, and KIRP showed negative correlation (r<-0.2). Further analysis with ESTIMATE algorithm showed the LGG, GBM, THCA, ACC and UVM were top 5 cancers positively associated with ESTIMATE scores, immune scores and stromal scores, but the CHOL and LUSC were top 2 cancers negatively associated with ESTIMATE scores, immune scores and stromal scores (**Figure 5B**). The correlations in LGG, GBM, CHOL and non-small cell lung cancer (NSCLC) were validated with GEO datasets (**Supplementary Figure 5**). Taken LGG, GBM, LUSC and CHOL as examples, we find the AKR1B10 expression is elevated in purity low group, stromal score high group, immune score high group and ESTIMATE score high group in LGG and GBM (**Figure 5C**); AKR1B10 expression is also elevated in the purity high group, stromal score low group, immune score low group and ESTIMATE score low group in LUSC and CHOL (**Figure 5C**). Together these data may imply the potential role of AKR1B10 in TME.

### 3.4 Potential role of ARK1B10 in immune responses

**Figure 6:**
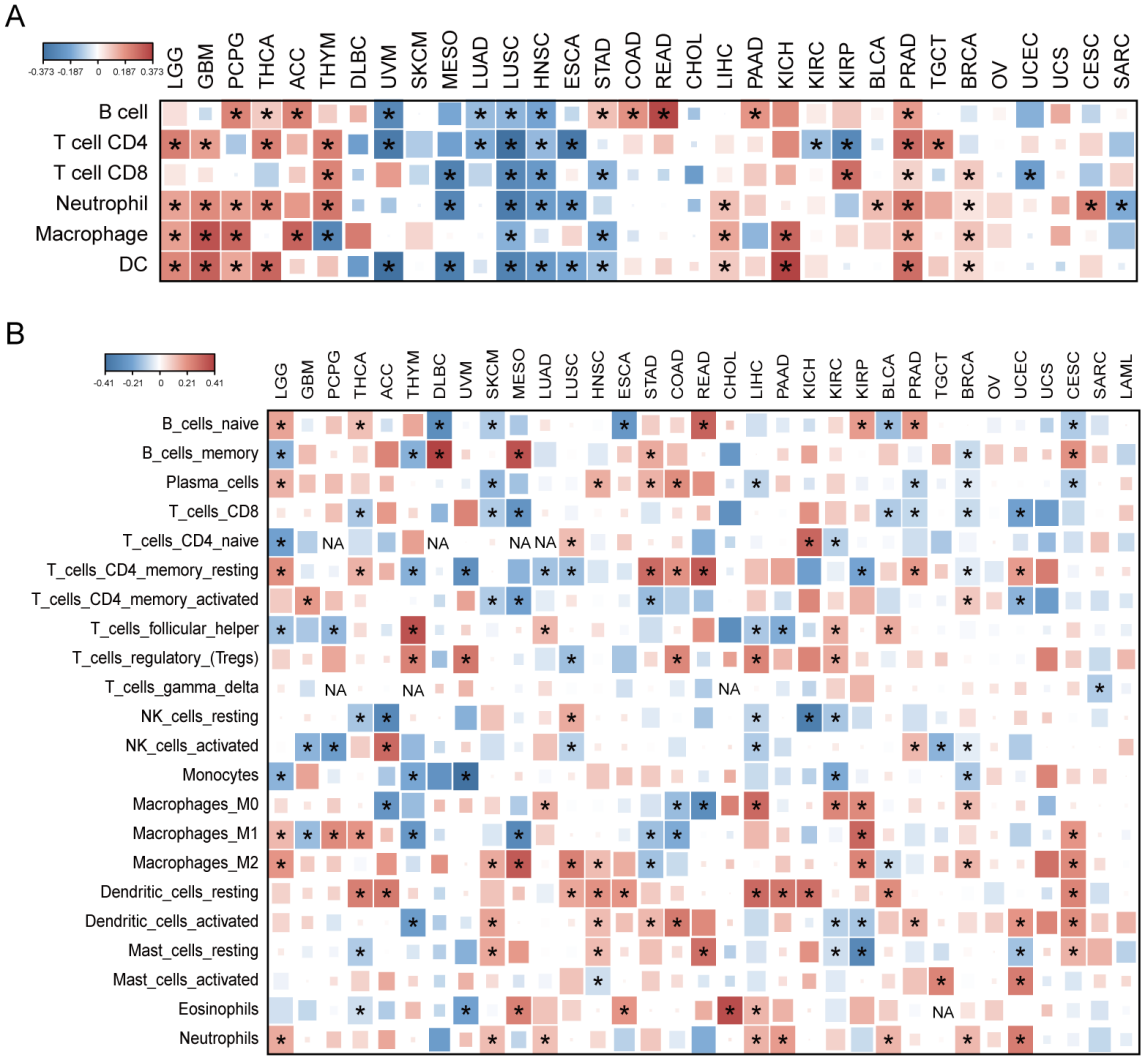
The correlation of AKR1B10 expression with immune cell types. Heatmap of AKR1B10 correlation with various immune cell types by TIMER **(A)** and CIBERSORT **(B)** algorithms, color represents the correlation efficiency, and the asterisk represents *p* < 0.05, NA means data not available.

**Figure 7:**
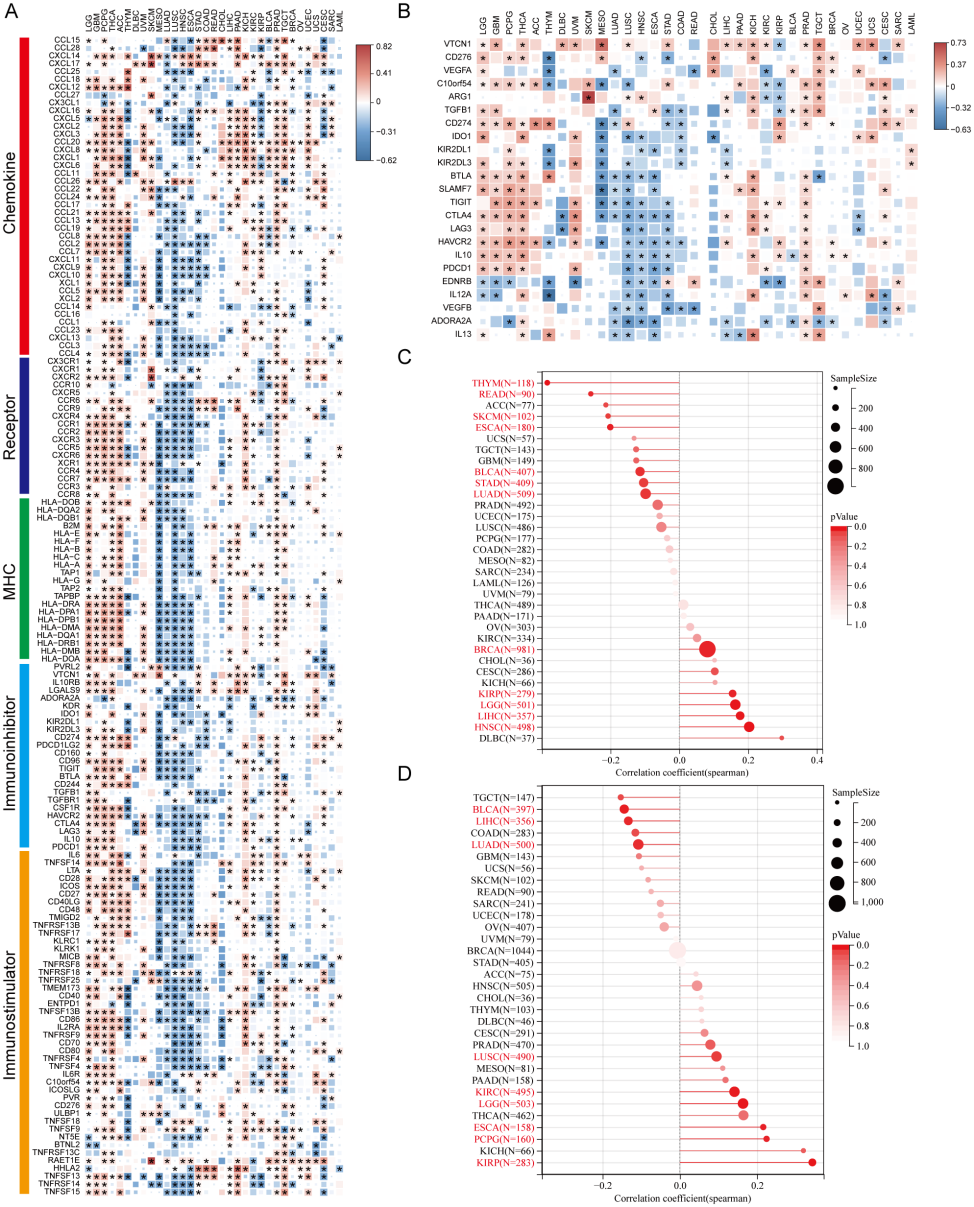
The correlation of AKR1B10 expression with immune related gene expression. Heatmap of AKR1B10 correlation with genes involved in 5 immune pathways **(A)**, and inhibitory immune checkpoints **(B)** in pan cancer, color represents spearman correlation efficiency, asterisk represents *p* < 0.05, NA means data not available. Lollipop plot of AKR1B10 correlation with TMB **(C)**, with HRD **(D)** in cancers, size of plot represents sample size of each cancer, color of plot represents *p* value, and each cancer with *p*<0.05 is labeled in red font.

We analyzed AKR1B10 expression and immune cells infiltrations with both TIMER and CIBERSORT algorithms. TIMER analysis showed the AKR1B10 expression was associated with multiple immune cells, especially CD4+ T cell, neutrophils and dendritic cells, and these associations were positive in GBM, THCA, THYM and PRAD, but negative in UVM, MESO, LUSC (**Figure 6A**). CIBERSORT analysis showed that the association was mainly focused on T cells CD4+ memory resting, T cells regulatory (Tregs), and dendritic cell resting (**Figure 6B**). AKR1B10 exhibited a broad correlation with immunomodulators involved in 5 immune pathways, among which the correlation with MHC pathway was highest (**Figure 7A**). Our results also supported an association of AKR1B10 expression with dendritic cells and T cells, indicating that AKR1B10 may be involved in antigen presentation.

### 3.5 Potential role of AKR1B10 in immune therapy responses

The immune therapy based on immune checkpoint inhibitors (ICI) is a novel and promising anti-tumor therapy mode. Our analysis showed the correlation between AKR1B10 expression and various ICIs in LGG, GBM, PCPG, THCA, ACC, UVM, KICH, and TGCT, but no correlations in THYM, MESO, LUSC, ESCA, and CHOL (**Figure 7B**).

Tumor mutation burden (TMB) was defined as the number of gene mutations (e.g., base substitutions, insertions and deletions in coding area) per million bases in somatic cells. It is an indicator of mutation frequencies. The higher TMB was usually associated with the favored ICI response regardless of PD-L1 status in various types of tumors. Our analysis showed AKR1B10 expression was significantly associated with TMB in 12 types of cancers, with HNSC correlation coefficient larger than 0.2, THYM, SKCM, ESCA and READ correlation coefficient smaller than -0.2 (**Figure 7C**). Together, these results indicate that AKR1B10 may be a potential predictor and target of immune therapies.

Homologous recombination deficiency (HRD) refers to HRR dysfunction caused by HRR related gene mutations or epigenetic inactivation. HRD often presents in various tumor and produces specific, quantifiable and stable genomic alterations. Clinical research showed HRD status was related to the sensitivity of PARP inhibitors and platinum chemotherapy. We found that AKR1B10 expression significantly correlated with HRD in 9 types of cancer, with ESCA, KIRP and PCPG correlation coefficient lager than 0.2 (**Figure 7D**).

### 3.6 Functional analysis of AKR1B10 in pan-cancer cells

**Figure 8:**
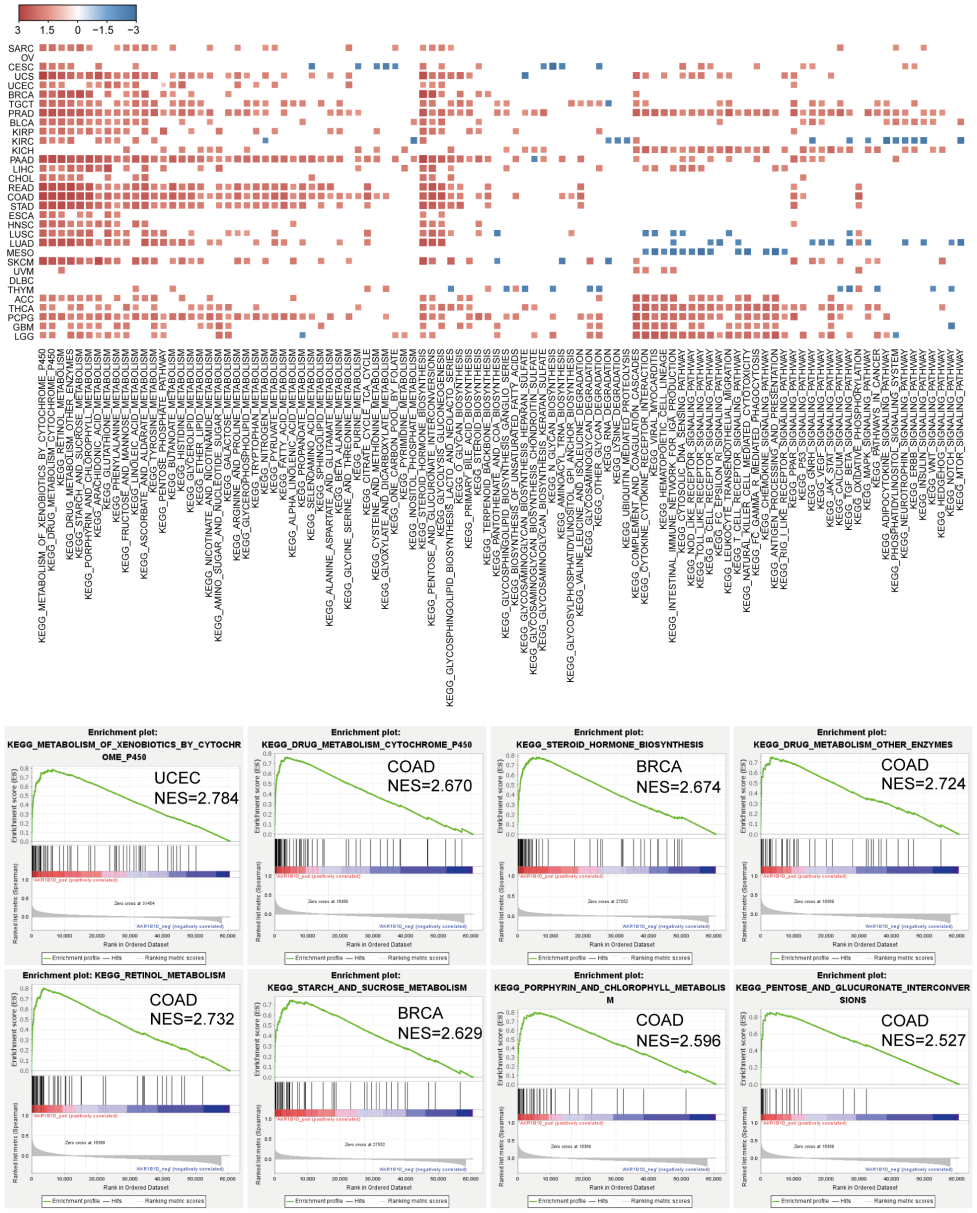
AKR1B10 enriched in metabolism associated KEGG pathway in cancers by GSEA. Heatmap showing AKR1B10 enriched in metabolic pathways in pan cancer, the color represents NES of GSEA analysis. Single GSEA plot shows top enriched pathway base on KEGG terms.

We further explored AKR1B10 function in pan-cancer cells through GSEA. We noticed that AKR1B10 was enriched with cancer cell features and pathways, as well as apoptosis, cell cycle, autophagy, and ECM interaction processes in some cancers. Further studies revealed that AKR1B10 was most remarkably and positively enriched with multiple metabolism processes, including drugs, hormones, saccharides, and amino acids in pan-cancer (**Figure 8**). These results indicate a potential role of AKR1B10 in cancer development and progression.

### 3.7 The prognostic role of AKR1B10 in pan-cancers

**Figure 9:**
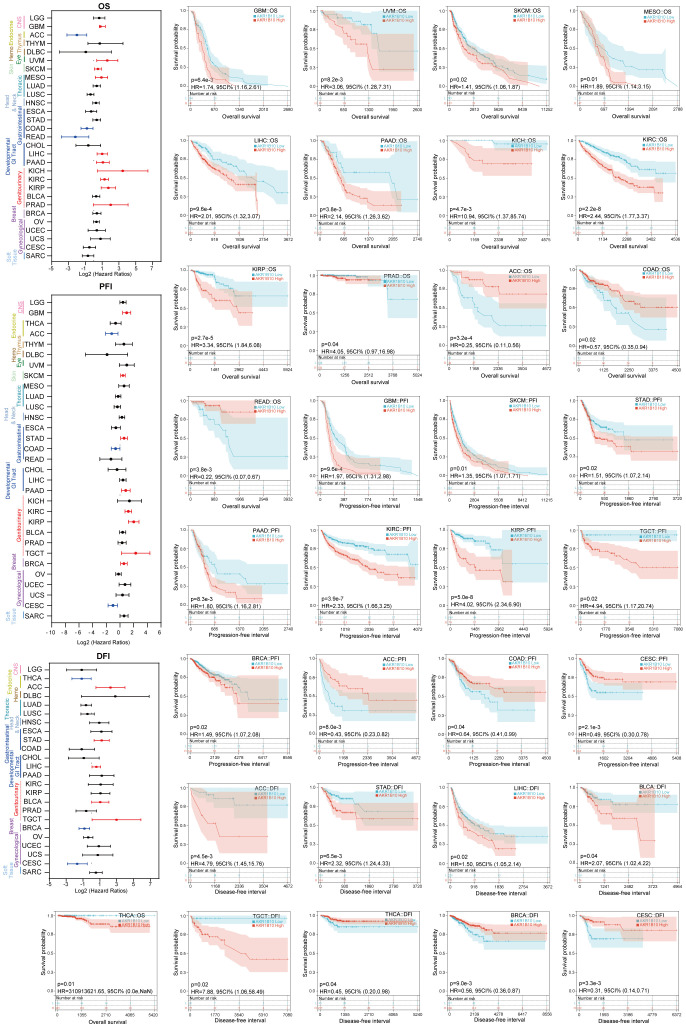
The correlation of AKR1B10 mRNA levels with progression in various types of cancers. Forest plot of AKR1B10 prognostic value in pan cancer, black lines represent not significant (*p* > 0.05), red lines represent a significantly poor association (Hazard Ratio > 1, *p* < 0.05), blue lines represent a significantly favored association (Hazard Ratio < 1, *p* < 0.05). Survival analysis of AKR1B10 in each cancer.

Subsequently, we evaluated the prognostic value of AKR1B10 in multiple types of cancers through Kaplan-Meier analysis. As shown in **Figure 9**, the increased AKR1B10 expression was significantly related to poor overall survival (OS) in GBM, THCA, UVM, SKCM, MESO, LIHC, PAAD, KICH, KIRC, KIRP, and PRAD, but better OS in ACC, COAD, and READ. In addition, elevated AKR1B10 expression was associated with shorter PFI in GBM, SKCM, STAD, PAAD, KIRC, KIRP, TGCT, and BRCA, as well as decreased DFI in ACC, STAD, LIHC, BLCA, and TGCT. On the contrary, increased AKR1B10 expression was associated with longer PFI in ACC, COAD, and CESC, and also the prolonged DFI in THCA, BRCA, and CESC.

### 3.8 Validation of AKR1B10 expression and its association with immune in gastric cancer

**Figure 10:**
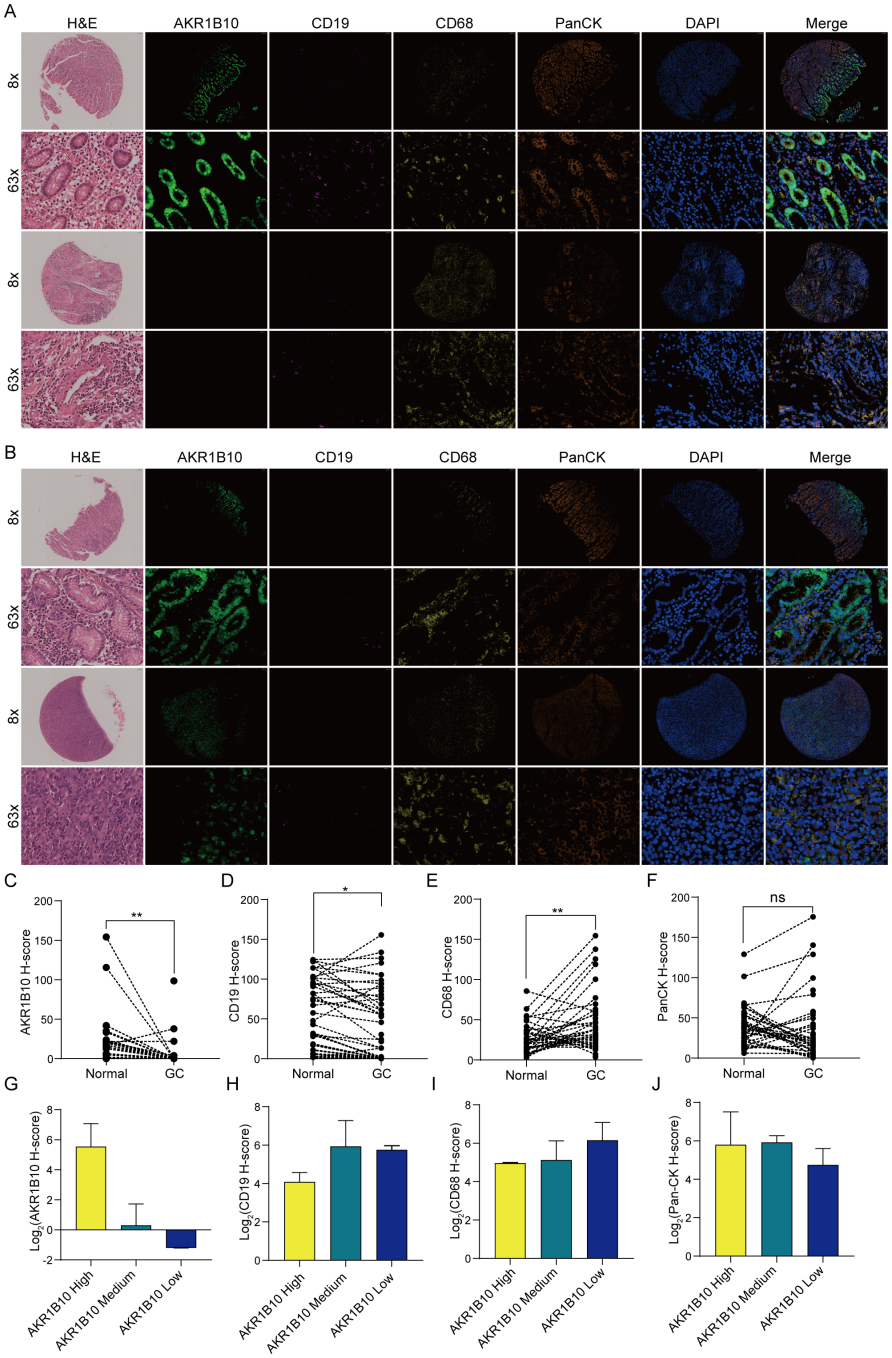
Immunohistochemistry of AKR1B10 in paired normal and gastric cancer. Representative picture of mIHC staining in paired normal and gastric cancer tissues **(A, B)**. Statistical analysis of AKR1B10 **(C)**, CD19 **(D)**, CD68 **(E)**, PanCK **(F)** H score in paired normal and gastric tissue. Statistical analysis of AKR1B10 **(G)**, CD19 **(H)**, CD68 **(I)**, PanCK **(J)** expression in gastric cancer tissue with AKR1B10 high, medium and low group. ns means not significant, * *p*<0.05, ** *p*<0.01.

Our previous analysis showed stomach was the organ with highest AKR1B10 expression. Some studies indicated that decreased AKR1B10 expression promote M2 macrophage marker expression in THP1 macrophage cells (46), promote NK cell kill activity on liver cancer cell Huh7 (47), and knock down of AKR1B8 transgenic mice (homologous of AKR1B10 in mice) show a immunity disorder in colon (48), however, the correlation between AKR1B10 expression and immune infiltration in STAD remains elusive. We evaluated AKR1B10 expression in paired normal and gastric cancer tissues, and the correlation of AKR1B10 expression with immune cells in gastric cancer (**Figure 10**). The results showed that AKR1B10 expression was dramatically decreased in gastric cancer compared to normal tissues (**Figures 10A, C**). Meantime, the CD19 expression (B cell marker) was also decreased in gastric cancer (**Figures 10A, D**), and CD68 (Macrophage marker) expression elevated in gastric cancer (**Figures 10A, E**). The PanCK (Epithelial marker) expression difference is not significant between gastric cancer and normal tissue (**Figures 10A, F**).

As AKR1B10 expression almost diminished in gastric cancer, we evaluated the relationship between AKR1B10 and immunity by dividing samples with AKR1B10 expression into AKR1B10 high group with H score > 10, AKR1B10 medium group with 10 > H score > 0.5 and AKR1B10 low group with H score < 0.5. We found CD68 show an elevated trend as AKR1B10 expression decreased (**Figures 10G, I**), which is consistent with the TIMER analysis (**Figure 6A**). However, either expression difference of CD19 or PanCK is clear (**Figures 10H, J**).

## 4 Discussion

In physiological conditions, AKR1B10 eliminates various endogenous and exogenous cytotoxic carbonyl compounds and mediates long-chain fatty acid/lipid synthesis, playing an important role in intestinal hemostasis. However, the role of AKR1B10 in different cancers is complex and remains elusive. In this study, we systematically analyzed the expression pattern, genetic alterations, and epigenetic alterations in 33 cancer types. We evaluated the association of AKR1B10 expression with components of tumor microenvironment and clinical outcomes. Our study provides a landscape of AKR1B10 in pan-cancers and suggests a potential role of AKR1B10 in tumor microenvironment remodeling. This study also takes gastric cancer as a role model to examine the AKR1B10 expression and its association with TME.

In literature, AKR1B10 mRNA was detected at a high level in adrenal gland and intestinal tract (11), and the AKR1B10 protein was expressed in small intestine, colon, and liver (1). AKR1B10 was reported to be upregulated in liver cancer (23, 49–51), lung cancer (27, 28), breast cancer (25), oral cancer (30–32), pancreatic cancer (52), cervical cancer (53) and papillary renal cell carcinoma (54). AKR1B10 was reported to suppress tumor development and progression in colorectal cancer (33–37) and gastric cancer (55), but remains controversial in nasopharyngeal cancer (56, 57) and esophageal cancer (58, 59). In this pan-cancer analysis we found that AKR1B10 is increased in GBM, LUAD, LUSC, CHOL, LIHC, PAAD, KIRP, UCEC and CESC, but decreased in STAD, COAD, READ, KICH, BLCA and PRAD. However, the BRCA and HNSC (including oral cancer and nasopharyngeal cancer) didn’t show significant alterations in AKR1B10 expression, which suggests the analysis with a refined cancer subtype may be helpful to clarify the AKR1B10 expression in special pathological conditions. As for prognostic values, AKR1B10 expression was reported to be correlated with poor prognosis in liver cancer (50), oral cancer (30–32), breast cancer (25), lung cancer (29), but with favored prognosis in CRC (34, 35). This pan-cancer analysis suggested that AKR1B10 also associated with poor prognosis in GBM, SKCM, STAD, PAAD, KIRC, KIRP, and TGCT, but with favored prognosis in ACC and CESC.

Tumor microenvironment underlies the basis of tumor growth, which contains kinds of cell and molecular components, forming a complex interaction net. Although the single cell RNA-Sequencing revealed AKR1B10 expression was mainly restrained in epithelial cells, we found that AKR1B10 expression was significantly associated with tumor purity, stromal cells, various immune cells infiltration (e.g., neutrophils, dendritic cells and CD4+ T cells) and immune gene expression. Cancer-associated-fibroblasts (CAF) were the main component of stroma, and participated in bidirectional regulation of tumorigenesis (60). Immune cells like CD4+ T cells and neutrophils were also reported to be dual functional in tumor immunity. CD4+ T cells contains a few subpopulations including Th1, Th2, Th9, Th17, follicular helpher T (TFH) cells and T cells regulatory (Treg). Among them, stable Tregs, which express foxp3, suppress immune responses by preventing Tconv cell activation, inducing B cell apoptosis, and restraining the antigen-presentation of DCs, while fragile Tregs exert an antitumor effect through secreting IFN-γ, and unstable Tregs was not suppressive (61). Tumor-associated neutrophils (TANs) are an important component of TME as well. Two types of TANs are identified in TME. The antitumor N1 kills tumor cells via oxidative stress and inflammatory factors. Pro-tumor N2 promotes tumor growth and dissemination via remodeling TME or forming neutrophils extracellular traps (NETs) (62). Neutrophils are novel potential targets of anti-cancer therapy (63). Dendritic cells (64) and immune checkpoints (65) both play crucial roles in immune responses, and are dazzling stars in tumor immunotherapy. One of the possible mechanisms linking AKR1B10 expression in epithelial cells with TME components may be the metabolism re-programming. For example, AKR1B10 may create an immunosuppressive TME by promoting long-chain fatty acid/lipid synthesis. These results suggest that AKR1B10 may be a promising regulator of TME immune status. Interestingly, our data showed for the first time that AKR1B10 expression was significantly associated with DNA methylation in pan-cancers. Further study is warranted.

This study verified AKR1B10 expression and immune infiltration in gastric cancer, and our results consistently demonstrated that AKR1B10 expression was diminished in gastric cancer accompanied with CD19+ B cell proportion decline and CD68+ macrophage increase. However, the function and prognostic value of AKR1B10 is still obscure, and the relationship with immune infiltration is largely unknown. For example, Yao et al. report that high AKR1B10 expression is associated with smaller tumor size, less metastasis and high survival rate of gastric cancer (55), but Ahmed et al. exhibit that high AKR1B10 is correlated with high metastasis, tumor progression, poor responses to chemotherapy and low survival rate in gastric cancer (66). Two independent studies show AKR1B10 may induce drug resistance of gastric cancer (67, 68), but the other report that AKR1B10 inhibit proliferation and migration of gastric cancer cells (69). The B cells are major components of tertiary lymphoid structures in gastric cancer, and associated with favored prognosis (70). Tumor associated macrophage contains M1 (anti-tumor) and M2 (pro-tumor) macrophage, deletion of AK1B10 results in a decrease to M1 polarization markers and an increase to M2 polarization markers (46). Considering the essential biological function of AKR1B10 and its downregulation in gastric cancer, we would like to consider AKR1B10 as a protective factor in normal gastric tissues. It is possible that AKR1B10 is important for the gastric cancer development, and relate to macrophage infiltration.

## 5 Conclusion

This study provided a landscape of AKR1B10 in pan-cancers, including its expression and potential roles in TME, tumor immunity, and survival. The data suggested that AKR1B10 may be a potential biomarker and therapeutic target for multiple cancers. This study particularly revealed the diminished expression of AKR1B10 in gastric cancer, and discussed the potential role of AKR1B10 in the gastric cancer TME.

## Data Availability

Publicly available datasets were analyzed in this study. This data can be found here: https://portal.gdc.cancer.gov/, GDC portal; https://www.proteinatlas.org/, HPA; http://bjs.zju.edu.cn/HCL/, HCL.

## Supplementary material

Supplementary Table 1 Description of cell type for HCL single cell analysis.
