# Supplementary material for: The pan-cancer landscape of aldo-keto reductase1B10 reveals that its expression is diminished in gastric cancer
Source: Front Immunol. 2024 Dec 6;15:1488042. doi: 10.3389/fimmu.2024.1488042 (PMC11659136; doi:10.3389/fimmu.2024.1488042)
Supplement: Supplementary file 1 [file DataSheet1.docx]

**
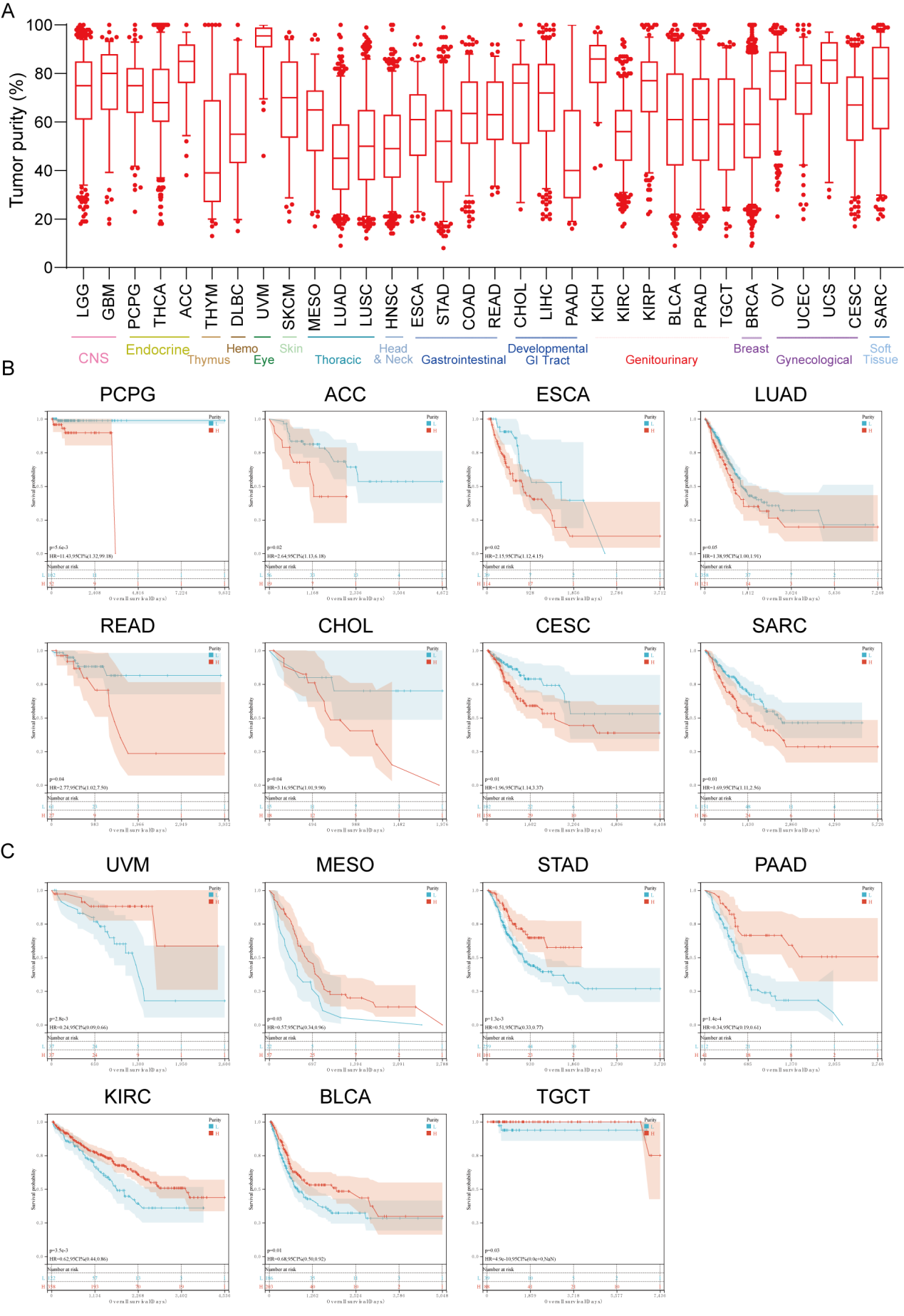
**

**Supplementary figure1. Tumor purity in pan-cancer and and its relationship with overal survival.** Box plot of tumor purity in pan-cancer (A). Kaplan–Meier survival plot show high purity correlates with poor prognosis in 8 types of cancer (B). Kaplan–Meier survival plot show high purity correlates with favored prognosis in 7 types of cancer (C).

**
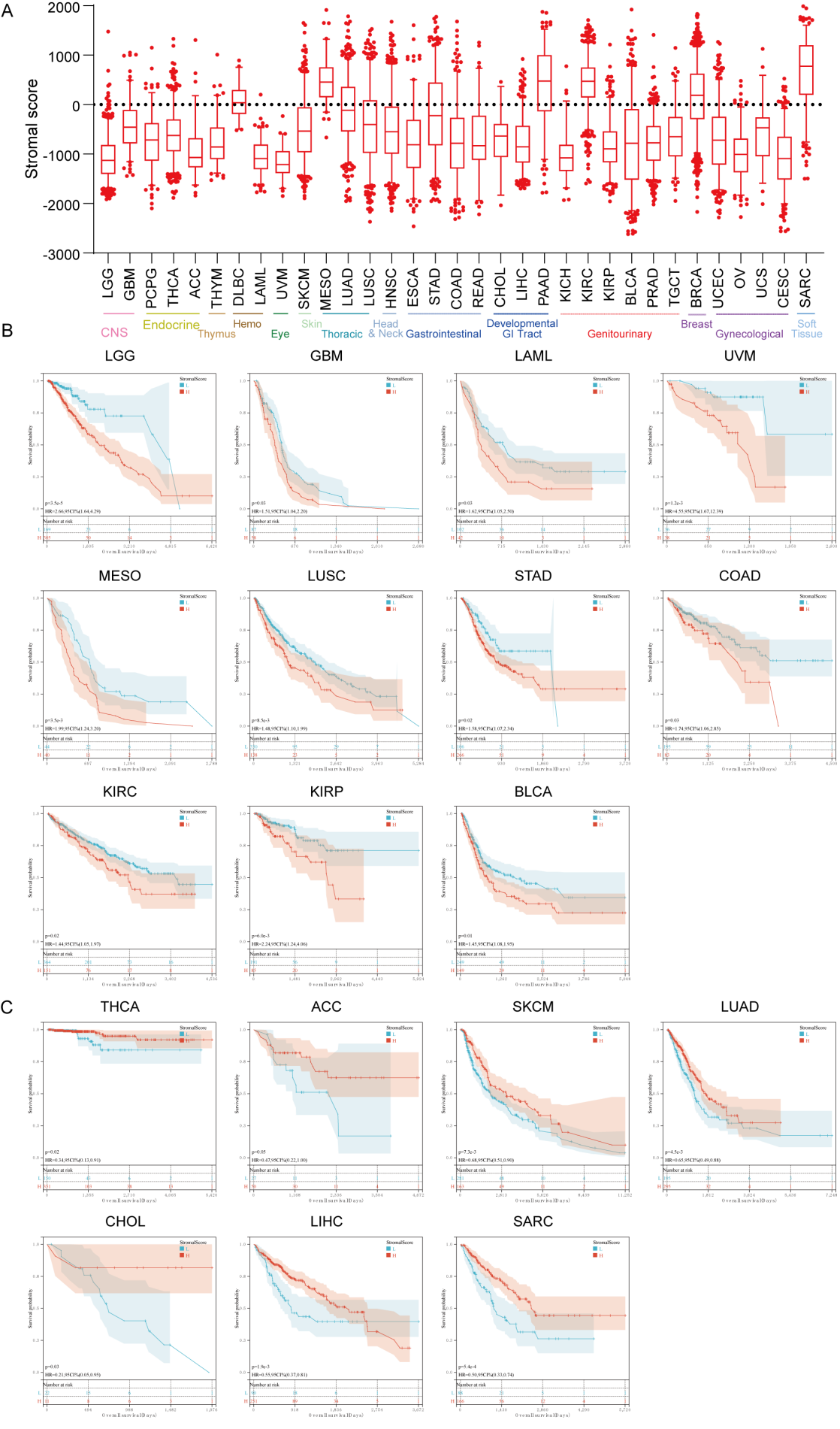
**

**Supplementary figure2. Stromal score in pan-cancer and and its relationship with overal survival.** Box plot of stromal score in pan-cancer (A). Kaplan–Meier survival plot show high stromal score correlates with poor prognosis in 11 types of cancer (B). Kaplan–Meier survival plot show high stromal score correlates with favored prognosis in 7 types of cancer (C).

**
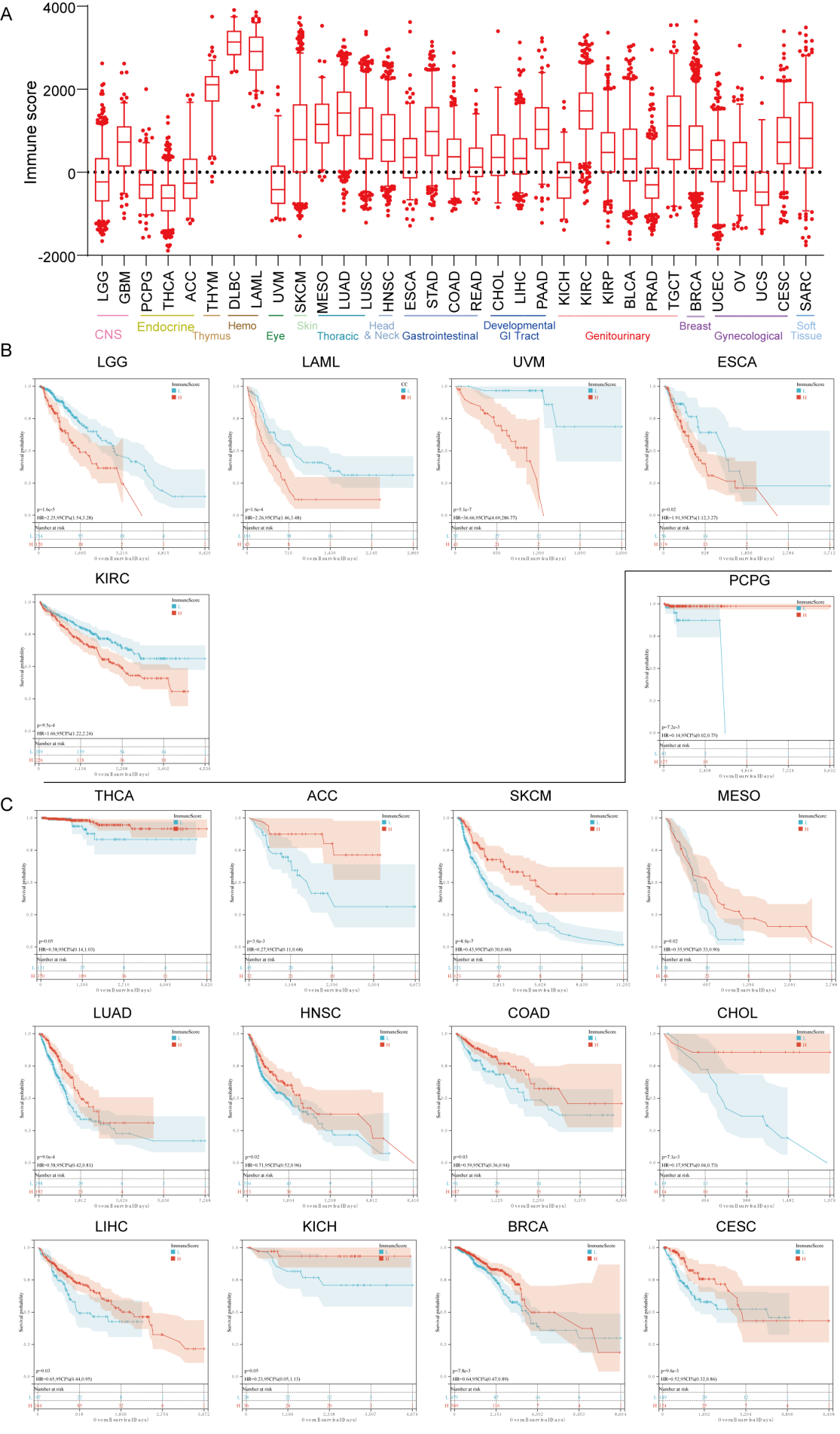
**

**Supplementary figure3. Immune score in pan-cancer and and its relationship with overal survival.** Box plot of immune score in pan-cancer (A). Kaplan–Meier survival plot show high immune score correlates with poor prognosis in 5 types of cancer (B). Kaplan–Meier survival plot show high immune score correlates with favored prognosis in 13 types of cancer (C).

**
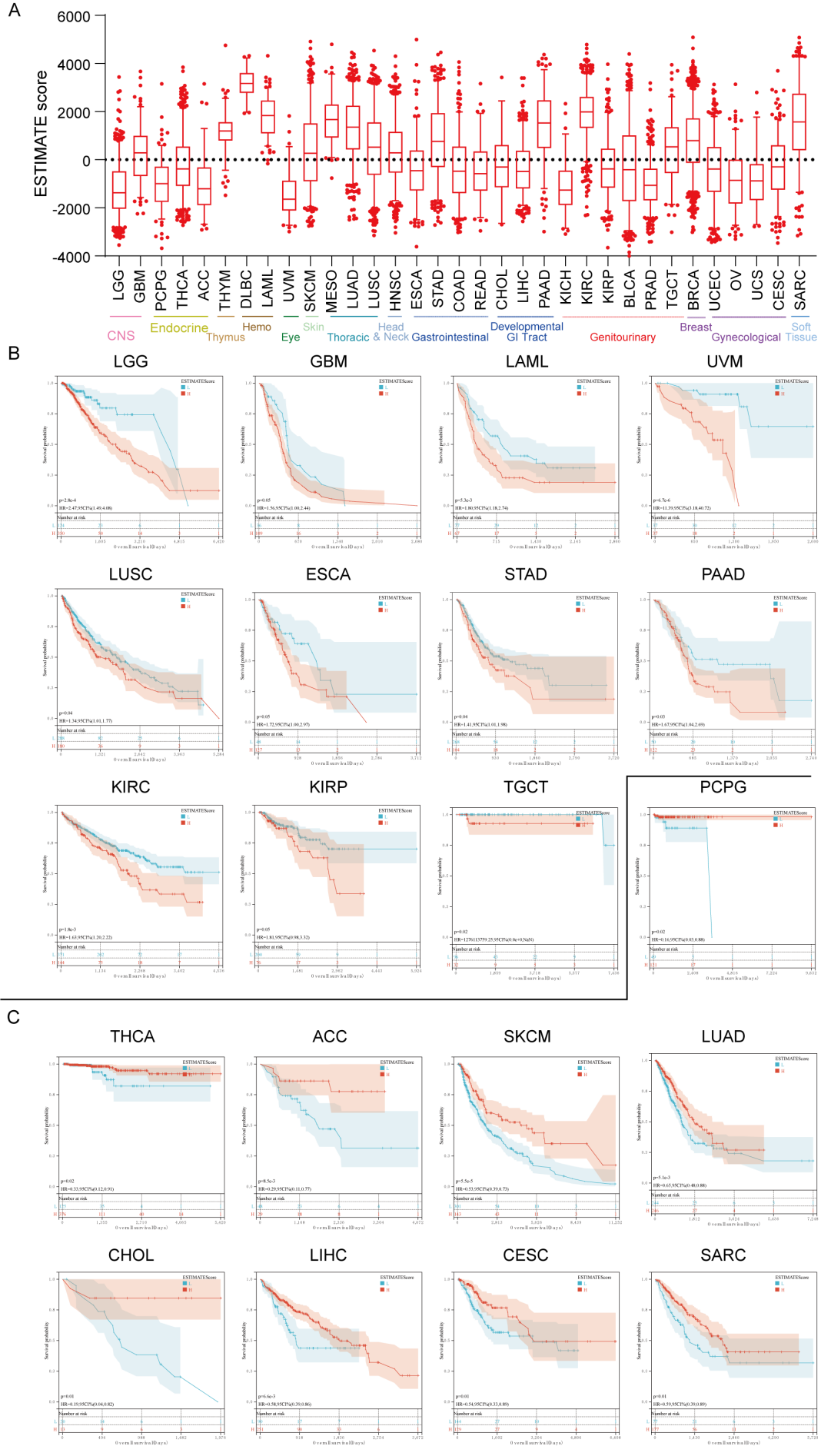
**

**Supplementary figure4. ESTIMATE score in pan-cancer and and its relationship with overal survival.** Box plot of ESTIMATE score in pan-cancer (A). Kaplan–Meier survival plot show high ESTIMATE score correlates with poor prognosis in 11 types of cancer (B). Kaplan–Meier survival plot show high ESTIMATE score correlates with favored prognosis in 9 types of cancer (C).

**
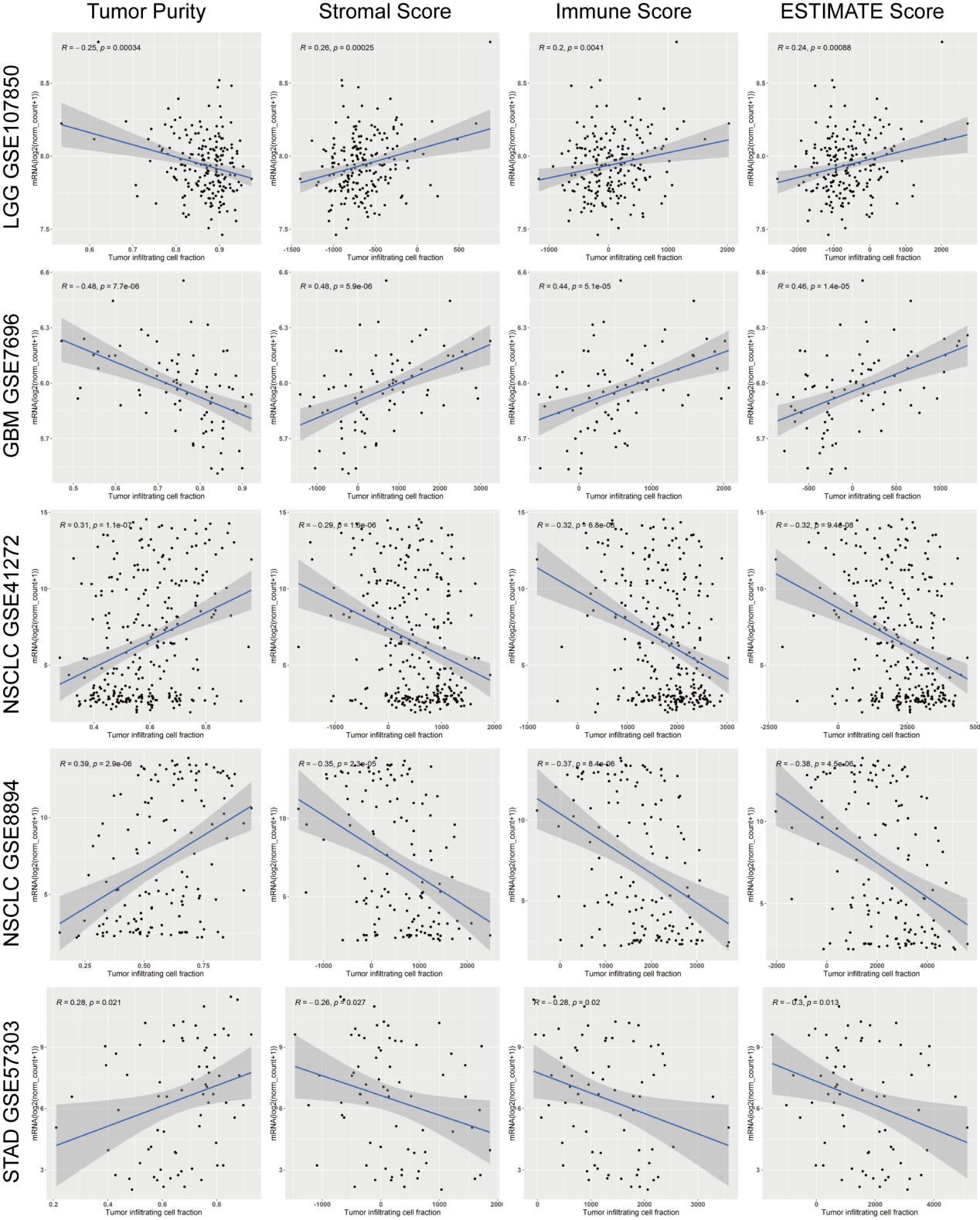
**

**Supplementary figure5. Validation of the correlation between AKR1B10 expression and computational tumor purity in GEO datasets.**
