# Supplementary material for: The pan-cancer landscape of aldo-keto reductase1B10 reveals that its expression is diminished in gastric cancer
Source: Front Immunol. 2024 Dec 6;15:1488042. doi: 10.3389/fimmu.2024.1488042 (PMC11659136; doi:10.3389/fimmu.2024.1488042)
Supplement: Supplementary Table 1 — Description of cell type for HCL single cell analysis. [file Table1.docx]

Supplementary table1. Description of cell type for HCL single cell analysis.

| Label | Cell type | Label | Cell type | Label | Cell type |
| --- | --- | --- | --- | --- | --- |
| C1 | Fetal epithelial progenitor | C35 | Smooth muscle cell | C69 | Macrophage |
| C2 | Macrophage | F36 | Fibroblast | C70 | Fibroblast |
| C3 | B cell (Plasmocyte) | C37 | B cell | C71 | Fetal acinar cell |
| C4 | Fibroblast | C38 | Epithelial cell | C72 | Stromal cell |
| C5 | Fasciculate cell | C39 | Enterocyte | C73 | Basal cell |
| C6 | T cell | C40 | Neutrophil (RPS high) | C74 | Ventricle cardiomyocyte |
| C7 | Fetal mesenchymal progenitor | C41 | Antigen presenting cell  (RPS high) | C75 | Erythroid cell |
| C8 | Endothelial cell (APC) | C42 | Smooth muscle cell | C76 | Mesothelial cell |
| C9 | Enterocyte progenitor | C43 | Fetal chondrocyte | C77 | Ureteric bud cell |
| C10 | Fetal stromal cell | C44 | Pancreas exocrine cell | C78 | Macorphage |
| C11 | Fetal neuron | C45 | Macrophage | C79 | Smooth muscle cell |
| C12 | Erythroid progenitor cell  (RP high) | C46 | Neutrophil | C80 | Enterocyte |
| C13 | Monocyte | C47 | M2 Macrophage | C81 | Epithelial cell |
| C14 | B cell (Plasmocyte) | C48 | Neutrophil | C82 | Stratified epithelial cell |
| C15 | Fetal enterocyte | C49 | Epithelial cell | C83 | Proximal tubule progenitor |
| C16 | Hepatocyte/ Endodermal cell | C50 | Gastric chief cell | C84 | Gastric endocrine cell |
| C17 | Fetal fibroblast | C51 | Macrophage | C85 | Erythroid cell |
| C18 | Fibroblast | C52 | Proliferating T cell | C86 | Mast cell |
| C19 | Fetal mesenchymal progenitor | C53 | Fetal neuron | C87 | hESC |
| C20 | Endothelial cell | C54 | Goblet cell | C88 | Fetal endocrine cell |
| C21 | Fetal stromal cell | C55 | Fasciculate cell | C89 | Neutrophil |
| C22 | Dendritic cell | C56 | Intercalated cell | C90 | Goblet cell |
| C23 | CB CD34+ | C57 | Stratified epithelial cell | C91 | Goblet cell |
| C24 | T cell | C58 | Stromal cell | C92 | Immature Sertoli cell  (Pre-Sertoli cell) |
| C25 | Unknow | C59 | Epithelial cell | C93 | Myeloid cell |
| C26 | Erythroid cell | C60 | Epithelial cell (Intermediated) | C94 | Dendritic cell |
| C27 | Stromal cell | C61 | Sinusoidal endothelial cell | C95 | Stromal cell |
| C28 | Oligodendrocyte | C62 | Primordial germ cell | C96 | Fetal Neuron |
| C29 | Endothelial cell | C63 | Fasciculate cell | C97 | Intermediated cell |
| C30 | AT2 cell | C64 | Fetal skeletal muscle cell | C98 | Epithelial cell |
| C31 | Monocyte | C65 | Enterocyte progenitor | C99 | Chondrocyte |
| C32 | Thyroid follicular cell | C66 | Endothelial cell (endothelial to mesenchymal transition) | C100 | B cell |
| C33 | Smooth muscle cell | C67 | Basal cell | C101 | Kidney intercalated cell |
| C34 | Fibroblast | C68 | Ventricle cardiomyocyte | C102 | Adrenal gland inflammatory cell |
